# Molecular characterization and novel genotypes of *Enterocytozoon bieneusi* in pet snakes in Beijing, China

**DOI:** 10.1016/j.ijppaw.2020.06.006

**Published:** 2020-06-17

**Authors:** Juanfeng Li, Dongfang Li, Haixia Zhang, Rongjun Wang, Zixiang Lin, Liwei Zhang, Yangwenna Cao, Meng Qi

**Affiliations:** a College of Animal Science, Tarim University, Alar, Xinjiang, 843300, China; b College of Animal Science and Veterinary Medicine, Henan Agricultural University, Zhengzhou, Henan, 450002, China; c College of Veterinary Medicine, China Agricultural University, Beijing, 100193, China

**Keywords:** *Enterocytozoon bieneusi*, Genotypes, ITS, Pet snakes, China

## Abstract

Little is known regarding the *Enterocytozoon bieneusi* genotypes in snakes worldwide. In the present study, a total of 273 fecal samples were collected from pet snakes in Beijing, China. They were then tested for the presence of *E. bieneusi* by PCR amplification of the internal transcribed spacer (ITS) gene. The overall infection rate of *E. bieneusi* was 4.4% (12/273), with the highest infection rate (20%, 1/5) of *E. bieneusi* was found in the Black rat snake (*Pantherophis obsoletus*), whereas no positive samples were detected from both Milk (0/22) and Coast garter snakes (0/2). Eight genotypes were identified, including four known genotypes: EbpA (n = 1), EbpC (n = 5), Henan-III (n = 1), and SHR1 (n = 1), and four novel genotypes: CRep-5 (n = 1), CRep-6 (n = 1), CRep-7 (n = 1), and CRep-8 (n = 1). Among them, EbpC (41.7%, 5/12) was the predominant genotype. Phylogenetic analysis showed that seven genotypes belonged to group 1, while genotype SHR1 belonged to group 2. Genotypes EbpA, EbpC, and Henan-III have been previously reported in humans. This suggests that pet snakes are a potential source of zoonotic microsporidiosis transmission in China.

## Highlights

• 4.4% (12/273) of pet snakes were PCR positive for *E. bieneusi.*
• Eight genotypes were identified, including four known and four novel genotypes.
• Transmission of *E. bieneusi* to humans from pet snakes is possible.

## Graphical abstract

**Figure:**
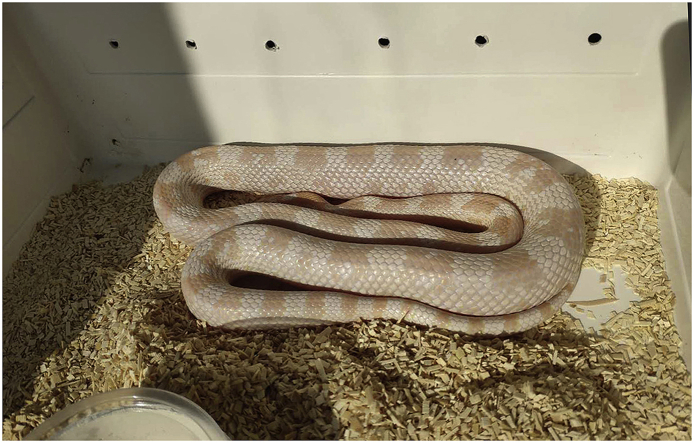

## 1 Introduction

*Enterocytozoon bieneusi*, an opportunistic intestinal pathogen associated with microsporidiosis. The parasite is widespread among humans, domestic animals, and wildlife in China (Li et al., 2019a). Transmission occurs through direct or indirect contact with contaminated food and water from infected humans or animals (Decraene et al., 2012). Self-limiting diarrhea, malabsorption, and wasting are the main clinical signs of *E. bieneusi* infection in healthy individuals, although life-threatening diarrhea in immunocompromised patients, especially in HIV^+^ persons, does occur (Wang et al., 2013).

Genotyping based on the internal transcribed spacer (ITS) region of the ribosomal RNA (rRNA) gene is a widely used approach in molecular epidemiology studies of *E. bieneusi* (Santin and Fayer, 2009; Deng et al., 2019). At present, over 500 genotypes have been classified into at least 11 groups (Li et al., 2019b). Among them, many of the common genotypes, such as D, EbpC, Type IV, Peru6, Peru8, and Peru11, are zoonotic, underscoring their importance in public health (Li et al., 2019c; Wang et al., 2018; Zhou et al., 2020).

Few studies have been carried out regarding the genetic characterization of *E. bieneusi* in snakes. In a previous study, six *E. bieneusi* genotypes, with two known (type IV and Henan V) and four novel genotypes (CRep-1 to CRep-4) were identified in captive snakes from the Guangxi province in China. These genotypes belong to the zoonotic group 1 (Karim et al., 2014a). The aim of the present study was to estimate the prevalence and molecular characterization of *E. bieneusi* from pet snakes in Beijing, China.

## 2 Material and methods

### 2.1 Ethics statement

Permission was obtained from the pet owners prior to collection of fecal samples. No specific permits were required for the described field studies. The pet owners were not engaged in any form of discussion about this study. The protocol was also reviewed and approved by the Ethics Committee of Tarim University.

### 2.2 Sample collection

A total of 273 fecal samples were collected from pet snakes belonging to 13 different owners. Snakes were kept in individual cages and the main food was mice. Fecal sample was collected individually from each snake using sterile disposable latex gloves and was transferred to individual plastic bags. Sex, breed, and owner were recorded. All fecal specimens were stored at 4 °C until the time of processing.

### 2.3 DNA extraction and PCR amplification

Genomic DNA was extracted from fecal samples (approximately 200 mg) using E.Z.N.A. Stool DNA kits (Omega Biotek Inc., USA) according to the manufacturer's instructions. The primers and thermal cycling parameters used for PCR amplification were used as previously reported by Sulaiman et al. (2003). The outer forward primer was 5′-GATGGTCATAGGGATGAAGAGCTT-3′, and the outer reverse primer 5′-AATACAGGATCACTTGGATCCGT-3′ was used. The nested forward primer 5′- AGGGATGAAGAGCTTCGGCTCTG-3′ and nested reverse primer 5′-AATATCCCTAATACAGGATCACT′ was used for the second round of amplification. The 2×EasyTaq PCR SuperMix (TransGene Biotech Co., Beijing, China) was used for PCR amplification. Positive (DNA from dairy cattle-derived genotype I) and negative controls (distilled water) were included in all PCR tests. Amplicons were resolved by electrophoresis on 1% agarose gels (w/v) using GelRed™ (Biotium Inc., Hayward, CA, USA) staining.

### 2.4 Sequencing and phylogenetic analysis

Positive secondary PCR products were sequenced by GENEWIZ (Suzhou, China). All products were sequenced in both directions to ensure accurate sequencing results. ClustalX 2.1 (http://www.clustal.org/) was used to align the resulting DNA sequences. Sequences were aligned with reference sequences downloaded from the National Center for Biotechnology Information (https://www.ncbi.nlm.nih.gov/) to determine genotypes.

Phylogenetic trees were constructed in MrBayes (Version 3.2.6, http://mrbayes.sourceforge.net/) with Bayesian inference (BI). Bayesian inference was conducted with four independent Markov chains run for 1,000,000 metropolis-coupled MCMC generations. The analysis was performed until the potential scale reduction factor approached 1, and the average standard deviation of split frequencies was <0.01. Phylograms were drawn using FigTree v.1.4 (http://tree.bio.ed.ac.uk/software/figtree).

The nucleotide sequences obtained in the present study were submitted to GenBank (https://www.ncbi.nlm.nih.gov/genbank/) under accession numbers MN736449-MN736456.

### 2.5 Statistical analyses

Differences in rates of infection among different ages and species were compared using chi-square tests in the software SPSS version 22.0. Results with a *P* < 0.05 were considered statistically significant.

## 3 Results and discussion

In the 273 fecal samples, 12 positive samples of *E. bieneusi*s (4.4%) were detected by PCR. A similar prevalence rate of 4.6% (11/240) has been reported in captive snakes in Guangxi province, China (Karim et al., 2014a). Furthermore, *E. bieneusi* infections were identified in pet snakes from 7/13 owners, with owner G having the highest infection rate (16.7%, 1/6) (Table 1). There was no significant association between *E. bieneusi* prevalence snake sex, although prevalence in males (8.3%) was higher than in females (3.0%) (*P* > 0.05) (Table 2). The highest infection rate was 20% (1/20) in black rat snakes (*Elaphe obsoleta obsoleta*). In contrast, *E. bieneusi* infection was not detected in either milk snakes (*Lampropeltis triangulum*) or coast garter snakes (*Thamnophis sirtalis*) (*P* > 0.05) (Table 2). Many factors contribute to the prevalence of *E. bieneusi,* such as geographical regions, the host health status, seasonal variations, and the sample size (Deng et al., 2019; Hu et al., 2017). Due to the limited reports of *E. bieneusi* in snakes, more research is needed to investigate whether there are differences in prevalence in snakes produced by different breeding methods or geographical distribution.

**Table 1:** Genotype distribution of *Enterocytozoon bieneusi* in pet snakes in Beijing.

Pet owner	No. positive/No. specimens (%)	Genotypes (n)
A	0/4 (0)	
B	2/50 (4.0)	CRep-5 (1), SHR1 (1)
C	0/21 (0)	
D	0/9 (0)	
E	1/31 (3.2)	EbpA (1)
F	0/23 (0)	
G	1/6 (16.7)	EbpC (1)
H	3/30 (10.0)	CRep-6 (1), EbpC (2)
I	2/28 (7.1)	EbpC (2)
J	0/17 (0)	
K	2/17 (11.8)	CRep-7 (1), CRep-8 (1)
L	0/9 (0)	
M	1/28 (3.6)	Henan-III (1)
Total	12/273 (4.4)	CRep-5 (1), CRep-6 (1), CRep-7 (1), CRep-8 (1), EbpA (1), EbpC (5), Henan-III (1), SHR1 (1)

**Table 2:** Genotypes distribution of *E. bieneusi* in different sex and breeding of pet snakes in Beijing.

Variable	No. positive/No. specimens (%)	Genotypes (n)
Sex		
Female	6/201 (3.0)	CRep-5 (1), CRep-8 (1), EbpA (1), EbpC (2), SHR1 (1)
Male	6/72 (8.3)	CRep-6 (1), CRep-7 (1), EbpC (3), Henan-III (1)
Species		
Black rat snake (*E. obsoleta obsoleta*)	1/5 (20.0)	EbpC (1)
Black Mexican king Snake (*L. getula nigrita*)	2/71(2.8)	CRep-5 (1), CRep-7 (1)
California king snake (*L. getulus californiae*)	2/24 (8.3)	EbpA (1), Henan-III (1)
Coast garter snake (*T. sirtalis*)	0/2 (0)	
Corn snake (*E. guttata guttata*)	1/66 (1.5)	SHR1 (1)
Hognose snake (*H. nasicus*)	5/70 (7.1)	CRep-6 (1), EbpC (4)
Milk snake (*L. triangulum*)	1/22 (0)	
Pine snake (*P. melanoleucus*)	1/13 (7.7)	CRep-8 (1)

Sequence analysis identified eight genotypes from 12 *E. bieneusi* positive samples, including four previously described (EbpA, EbpC, Henan-III, and SHR1) and four novel genotypes (CRep-5 to CRep-8) (Table 1). Among them, genotypes CRep-6 CRep-7 and CRep-8 are closely related to genotype EbpC (AF135832). These genotypes differ by just one (G→A at locus 226 of internal transcribed spacer), two (C→T at loci 93 and 209) and one (G→A at locus 197) single nucleotide polymorphisms (SNPs), respectively. Genotypes CRep-5 differs at one SNP (T→C at locus 90) compared with genotype Henan-IV (JQ029727) (Karim et al., 2015).

Genotype EbpC (41.7%, 5/12) was the preponderant genotype in the present study, and the remaining seven genotypes accounted for 1 sample each (Table 1). Furthermore, EbpA and EbpC were the most common genotypes identified in both humans and animals worldwide. Genotype EbpA has been reported in humans, pigs, rats, and bamboo rats (Fiuza et al., 2015; Li et al., 2019c, 2020; Wang et al., 2019). Genotype EbpC has been reported in humans, nonhuman primates, pigs, deer, goat, cattle, rats, and in environmental samples (Karim et al., 2014b; Li et al., 2019a, 2020; Udonsom et al., 2019; Xie et al., 2019). Genotype Henan-III, has been identified in humans, pigs and whooper swans (Li et al., 2014, 2019a; Wang et al., 2013, 2020), whereas SHR1 has been reported in experimental rats (Li et al., 2020). As pets come in close contact with humans, and pet snakes can harbor the zoonotic genotypes, the potential for snake owners to become infected with *E. bieneusi* is a real concern.

**Fig. 1:**
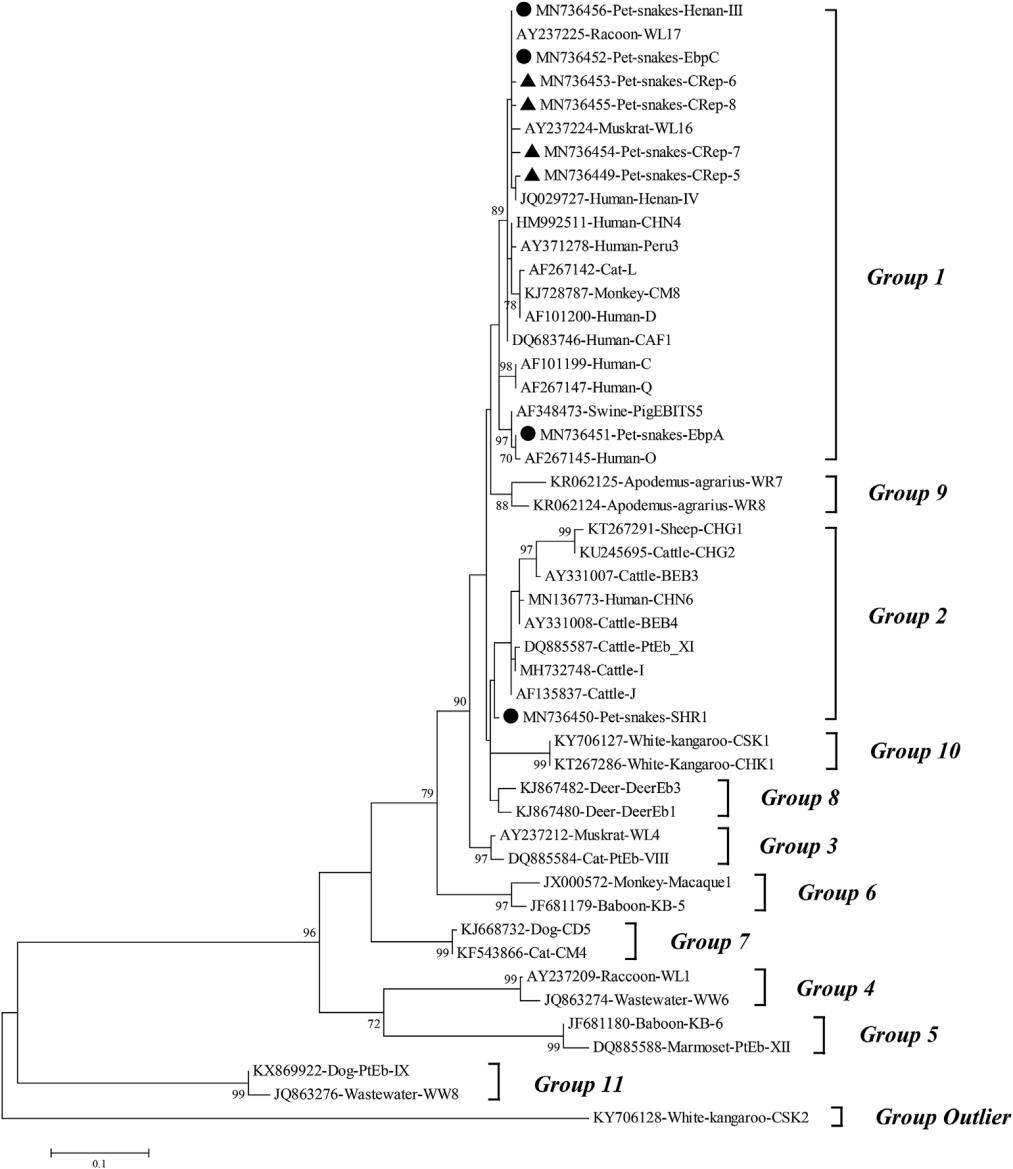
Phylogenetic relationships between the *E. bieneusi* genotypes identified in this study and other reported genotypes. The relationships were inferred using Bayesian inference analysis of the ITS gene sequences. Statistically significant posterior probabilities are indicated on the branches. Genotypes with filled circles and triangles are known and novel genotypes identified in this study, respectively.

Phylogenetic analysis showed that genotypes EbpA, EbpC, Henan-III and CRep-5 to CRep-8 clustered with group 1, while SHR1 belongs to group 2 (Fig. 1). Due to the low host specificity, groups 1 and 2 are considered to be zoonoses. In contrast, genotypes within groups three through 11 exhibit a tightly restricted host range (Li et al., 2019a). With more and more genotypes beingn identified, it is likely that additional groups will be added to *E. bieneusi.* Moreover, zoonotic *E. bieneusi* genotypes (e.g., D, EbpA, and EbpC) have been reported in rodents, including experimental mice and wild rats (Li et al., 2020). These animals make up the majority of the snake diet, especially those raised in captivity. Whether the source of *E. bieneusi* infection of snakes is foodborne or not has yet to be investigated.

In conclusion, *E. bieneusi* was low infection rate in pet snakes in Beijing in China. Of the 12 identified genotypes, seven are zoonotic, with genotype EbpC the predominant genotype. This indicates the potential of pet snakes as the possible vectors for the transmission of *E. bieneusi* to humans.

## Declaration of competing interest

All authors declare no conflicts of interest.
